# The Responses of Mouse Preimplantation Embryos to Leptin *In Vitro* in a Transgenerational Model for Obesity

**DOI:** 10.3389/fendo.2017.00233

**Published:** 2017-09-13

**Authors:** Martina Kšiňanová, Štefan Čikoš, Janka Babel’ová, Zuzana Šefčíková, Alexandra Špirková, Juraj Koppel, Dušan Fabian

**Affiliations:** ^1^Institute of Animal Physiology, Slovak Academy of Sciences, Košice, Slovakia

**Keywords:** leptin, maternal obesity, mouse model, preimplantation embryo, *in vitro* culture, apoptosis

## Abstract

The aim of the present study was to test the hypothesis that leptin can directly mediate the negative effect of maternal obesity on preimplantation embryos. As previously shown, maternal obesity retards early embryonic development *in vivo* and increases the incidence of apoptosis in blastocysts. When two-cell embryos isolated from control and obese mice were transferred to identical (leptin free) conditions *in vitro*, no differences in any growth or quality parameters were recorded, including apoptosis incidence in blastocysts. Embryos isolated from control mice responded to transfer to environments with a high concentration of leptin (10 ng/mL) with a significant increase in arrest at the first or subsequent cell cycle. However, the majority of non-arrested embryos developed into blastocysts, showing morphology comparable to those cultured in the leptin-free group. On the other hand, the exposure of embryos isolated from obese mice to high leptin concentration *in vitro* did not retard their development. Furthermore, these embryos developed into blastocysts, showing a lower incidence of apoptosis. *In vivo*-developed blastocysts recovered from obese mice showed elevated expression levels of the proapoptotic gene *BAX* and the insulin-responsive glucose transporter gene *SLC2A4*. In conclusion, elevated leptin levels have both positive and negative effects on preimplantation embryo development *in vitro*, a response that likely depends on the body condition of the embryo donor. Moreover, these results suggest that leptin acts as a survival factor rather than an apoptotic inductor in embryonic cells. Since no elevations in the expression of the leptin receptor gene (*LEPR*) or fat metabolism-associated genes (*PLIN2, SLC27A4*) were recorded in blastocysts recovered from obese mice, the role of leptin in mediating the effects of obesity on embryos at the peripheral level is likely lower than expected.

## Introduction

Leptin, a 16-kDa helical cytokine, is produced by the obese *LEP* (*OB*) gene and is primarily secreted from adipose tissue (1). Since the levels of this adipokine in circulation are a direct function of body fat mass, individuals with developed obesity typically show chronic hyperleptinemia, which, in time, could lead to the development of central and/or peripheral leptin resistance.

Moreover, leptin is not only a messenger of the amount of energy stores in the brain but also a crucial hormone for a number of diverse physiological processes. The hypothesis that this peptide plays an important role in reproduction stems from several findings, particularly the fact that leptin-deficient (*OB/OB*) mice are infertile and that their fertility can be restored by the exogenous provision of leptin (2). It is currently widely believed that a minimum level of leptin is required for the maintenance of reproductive function in a majority of mammalian species (3).

Although the actions of leptin in the control of reproductive function are thought to be exerted mainly via the hypothalamic–pituitary–gonadal axis, the capacity of leptin to induce direct effects at the peripheral level has also been suggested (4). Leptin is present in the follicular, oviductal, and uterine fluids of different species (5–9); thus, this cytokine is available to the oocyte and embryo. The expression of the leptin receptor, a single transmembrane protein and member of the gp130 family of cytokine receptors, occurring in six splice variants (LeptRa-f) (10), has also been documented in the germ and embryonic cells of several species [reviewed in Ref. (4, 11)]. In mouse preimplantation embryos, it was shown from the two-cell stage onward (12). However, previous data documenting the direct effect of leptin during early pregnancy are inconsistent. Experimental *in vitro* studies have documented that the presence of leptin in culture media might have a positive, a negative or no effect on the development and proliferative activity of preimplantation embryos in dependence on concentration and species [reviewed in Ref. (4, 11).]. Therefore, further evaluation of the role of leptin in appropriately designed biological experiments is necessary, particularly when obesity and obesity-related infertility are growing problems in both human and animal populations.

The aim of the present study was to test the hypothesis that leptin could be one of the direct mediators of the previously documented negative effects of maternal obesity on early embryonic development and the incidence of apoptosis in blastocysts (13, 14). Both apoptosis-inducing and apoptosis-inhibiting effects of leptin have been reported in various cell lineages: leptin increased the incidence of apoptosis in adipocytes or ovarian granulosa cells, although leptin also enhanced the survival of leukocytes, pancreatic, and endometrial cells [reviewed in Ref. (15)]. Both functions have also been documented in germ and embryonic cells (16–18).

First, we compared the developmental capacities of the two-cell embryos isolated from control and obese females under standard (maternal mediators lacking) *in vitro* conditions. A two-generation model based on the overnutrition of experimental animals during intrauterine and early postnatal development, previously developed in our laboratory (13), was employed to produce mice with an obesity-like phenotype. Obese mice, derived from dams fed a high-energy diet, regularly show significantly elevated body weight and body fat, with massive fat deposits in the abdominal and perirenal areas and display increased plasma glucose, insulin, and leptin levels (13, 19).

Subsequently, we evaluated the responses of two-cell embryos isolated from control and obese mice to an *in vitro* environment with an elevated concentration of leptin (10 ng/mL; reflecting a hyperleptinemic status in mouse mothers). The average plasma levels of leptin in normal control mice are variable and depend on age, sex, strain and type of diet (20). For example, while the blood concentration of leptin in 6-week-old C57Bl/J6 males was lower than 1 ng/mL (21), in 6-week-old control female mice of the CD-1 strain, blood leptin levels reached 1.25 ng/mL on average (13). When C57Bl/J6 mice were fed a high-fat diet for 4 weeks, the leptin levels doubled, and after 16 weeks on this same diet, leptin levels increased to values higher than 10 ng/mL (21). Progressive hyperleptinemia with values higher than 20 ng/mL was documented also in 16- to 25-week-old obese mice possessing the lethal yellow mutation (22).

Finally, we assessed the expression profiles of several genes associated with apoptotic processes and energy metabolism (including leptin and its receptor) in blastocysts developed in the reproductive tracts of naturally fertilized control and obese mice.

## Materials and Methods

### Animals and Experimental Design

All experiments were performed on outbred ICR (CD-1 IGS) mice. The mice were purchased from Velaz (Prague, Czech Republic) and were housed in the animal facility at the Institute of Animal Physiology, Košice, Slovakia (authorization n. SK UC 06016). All animal experiments were performed in accordance with the ethical principles of the Ethical Committee for Animal Experimentation at the Institute of Animal Physiology, approved through the State Veterinary and Food Administration of the Slovak Republic (Ro 2296/13-221c) in strict accordance with Slovak legislation based on EU Directive 2010/63/EU on the protection of animals used for experimental and other scientific purposes. All efforts were made to reduce animal suffering and distress and to minimize the number of animals necessary to produce reliable results.

Adult female mice of the parental generation (5 to 6 weeks old, Velaz) were synchronized with eCG (pregnant mare’s serum gonadotropin; 5 IU ip; Folligon, Intervet International, Boxmeer, Holland) and human chorionic gonadotropin (4 IU ip; Pregnyl, Organon, Oss, Holland; 47 h later) and were subsequently mated with males of the same strain (12 to 24 weeks old, Velaz) overnight. Mated dams were randomly divided into two groups: control and experimental. The animals in both groups were fed a standard pellet diet (M1, Řicmanice, Czech Republic, 3.2 kcal/g, with 225 g/kg of crude protein, 27 g/kg of crude fat, 30 g/kg of crude fiber, 61 g/kg of ash, and 58 g/kg of saccharides) *ad libitum*. The animals in the experimental group were additionally fed with Ensure Plus high-energy nutritional product (1.5 kcal/mL) *ad libitum*. To minimize the impact of nutrition on the reproductive process, after weaning, the female mice of the F1 generation delivered from both control and experimental dams were only fed a standard pellet diet (M1).

At an age of 34 days, body weight and body fat were measured in all females of the F1 generation. The mice were individually scanned using a non-invasive nuclear magnetic resonance instrument (Echo MRI, Whole Body Composition Analyzer, Echo Medical System, Houston, TX, USA). The females were placed in a specially sized, transparent plastic holder without sedation or anesthesia. The holder was subsequently inserted into a tubular space in the side of the EchoMRI system. The animals were free to move, and the time of individual scanning did not exceed 60 s.

Females delivered from dams fed a standard diet were allocated into two groups: (1) “normal” controls with physiologically normal body weight and body fat (7–8%) and (2) lean controls spontaneously displaying decreased body weight and body fat (<7%). Females delivered from dams fed a high-energy diet were classified as follows: (1) Obese mice with significantly elevated body weight and fat (>11%) and (2) obesity induction-resistant experimental mice with physiologically normal body weight and slightly elevated body fat (8–11%). In the following reproductive experiments, only control mice with physiological body weights and body fat (*n* = 148) and obese experimental mice with significantly elevated body weights and body fat (*n* = 177) were used.

At 35 days of age, spontaneously ovulating female mice were mated with males of the same strain (Velaz, 12 weeks old, fed standard diet, weight 38.49 ± 0.43 g) for one or more nights. Successful mating was confirmed by the identification of a vaginal plug every morning. Fertilized dams from both groups were sacrificed using cervical dislocation and were subjected to embryo isolation at the two-cell stage on day 2 of pregnancy (approximately 32 h after presumed ovulation, Table 1) or blastocyst isolation on day 4 of pregnancy (approximately 80 h after presumed ovulation, Table 2). Embryo collection was performed in six separate experiments.

**Table 1:** Somatic parameters of control and obese female mice and embryo production on day 2 of pregnancy in fertilized mice.

	Control mice	Obese mice
Female mice (*n*)	67	93
Average body weight (g)	19.04 ± 0.28^a^	24.32 ± 0.17^b^
Average body fat (% of body weight)	7.99 ± 0.05^a^	11.94 ± 0.11^b^
Fertilized embryo donors (*n*)	39	49
Average number of embryos per dam	10.85 ± 0.47	11.27 ± 0.43
Number of isolated embryos	423	552
Embryos at the two-cell stage (%)	91.25	89.49

*Results are expressed as the mean values ± SEM or as percentages. Different letters in superscript indicate a significant difference: body weight: Student’s *t*-test (*P* < 0.001); body fat: Mann–Whitney test (*P* < 0.001); number of embryos per dam: Mann–Whitney test (*P* > 0.05); embryos at the two-cell stage: chi-square test (*P* > 0.05)*.

**Table 2:** Somatic parameters of control and obese female mice and blastocyst production on day 4 of pregnancy in fertilized mice.

	Control mice	Obese mice
Female mice (*n*)	81	84
Average body weight (g)	18.96 ± 0.24^a^	22.65 ± 0.20^b^
Average body fat (% of body weight)	7.50 ± 0.07^a^	12.35 ± 0.17^b^
Fertilized embryo donors (*n*)	55	45
Average number of embryos per dam	9.62 ± 0.38	10.20 ± 0.44
Number of isolated blastocysts	385	359

*Results are expressed as the mean values ± SEM. Different letters in superscript indicate a significant difference: body weight: Student’s *t*-test (*P* < 0.001); body fat: Mann–Whitney test (*P* < 0.001); number of embryos per dam: Mann–Whitney test (*P* > 0.05)*.

Additionally, on day 4 of pregnancy, nine randomly selected females in each group were sacrificed through decapitation and subjected to blood collection. Serum prepared from the collected blood samples was stored at −80°C until the day of the assay. Leptin concentrations were quantified using a commercial ELISA kit (Mouse leptin 96-well plate assay; Millipore, Billerica, MA, USA), according to the manufacturer’s instructions.

### Isolation and Culture of Embryos

Embryos were recovered by flushing the oviduct (on day 2) or flushing both the oviduct and the uterus (on day 4) using a flushing-holding medium (23) and were subsequently classified under a stereomicroscope (Olympus SZ51, Japan).

Embryos at the two-cell stage isolated from dams of each condition type (control and obese, Table 1) were randomly divided into two subgroups and cultured *in vitro* under standard conditions (a humidified atmosphere with 5% CO_2_ and 37°C) for 72 h with/without the presence of recombinant mouse leptin expressed in *Escherichia coli* (#L3772, Sigma-Aldrich, Bratislava, Slovakia). To provide an appropriate developmental environment (i.e., one embryo per at least 1 µL of medium), 20 to 25 embryos were placed into 30 µL of synthetic oviductal medium KSOMaa Evolve containing an elevated potassium concentration, half-strength Eagle non-essential amino acid mixture (Zenith Biotech, Canada) and 0.1% (w/v) bovine serum albumin (BSA, Sigma-Aldrich). Drops of medium were placed in plastic cell culture dishes and covered with mineral oil (Zenith Biotech). According to the manufacturer’s instructions, leptin was dissolved in 20 mM TRIS/HCl (Sigma-Aldrich) at pH 8. The final concentration of leptin in KSOMaa was 10 ng/mL.

To test potential toxicity of leptin’s solvent, parallel culture experiments with the equivalent amount of 20 mM Tris/HCl (0.001% v/v) were provided (Datasheet S1 in Supplementary Material).

Blastocysts isolated from dams of each condition type (control and obese, Table 2) on day 4 of pregnancy were subjected to RNA extraction.

### Evaluation of *In Vitro*-Cultured Embryos

On the final day of culture (day 5), in each subgroup, the numbers of embryos reaching blastocyst, morula and lower stages of development (3- to 16-cell) and arrested (2-cell) and degenerated embryos (showing extensive cytoplasmic fragmentation) were assessed using stereomicroscopy.

**Figure 1:**
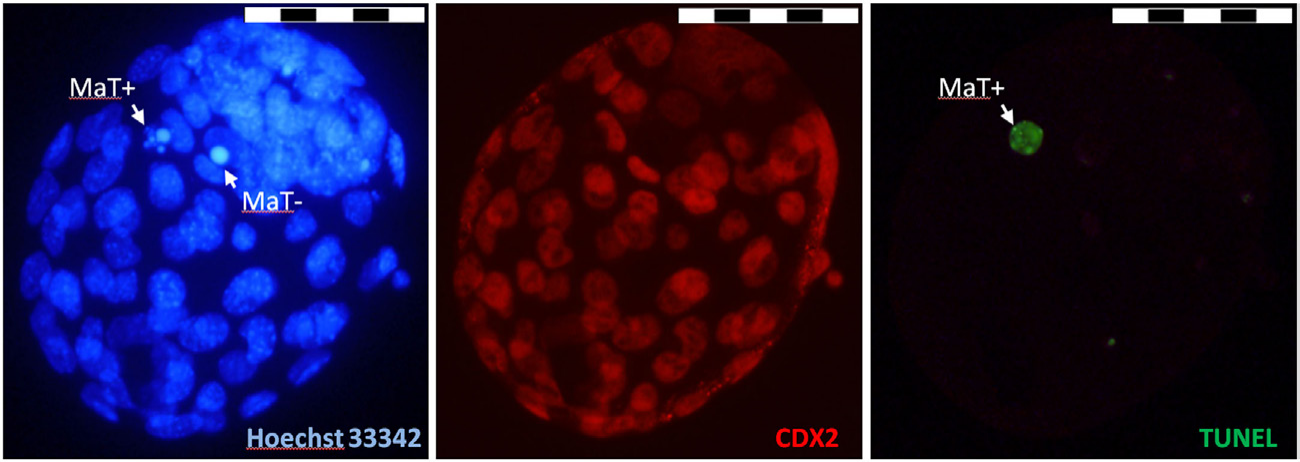
Illustrative fluorescence micrographs of mouse blastocyst obtained *in vitro*. Original magnification: ×400. Scale bar: 50 µm. The nuclear morphology was visualized by chromatin staining using Hoechst 33342 (blue), the trophectodermal (TE) cell lineage was visualized via the immunohistochemical labeling of CDX2 protein (red), and specific DNA degradation in the nucleoplasm was visualized through terminal deoxynucleotidyl transferase dUTP nick end labeling (TUNEL) labeling (green). MaT+: apoptotic cell with fragmented nuclear morphology and TUNEL-positive nucleoplasm; MaT−: apoptotic cell with condensed nuclear morphology and TUNEL-negative nucleoplasm.

For blastocysts obtained *in vitro*, the cell number, cell differentiation and cell death incidence were evaluated using immunochemistry, followed by fluorescence microscopy (Figure 1). The total number of nuclei in blastocysts and the nuclear morphology were assessed using Hoechst 33342 DNA staining. To identify the trophectodermal (TE) cell lineage, the immunohistochemical visualization of CDX2 protein was performed. The presence of specific DNA fragmentation was visualized using a terminal deoxynucleotidyl transferase dUTP nick end labeling (TUNEL) assay.

In detail, the blastocysts were fixed in 4% paraformaldehyde (Merck, Darmstadt, Germany) in phosphate-buffered saline (PBS) for 30 min at room temperature and stored in 1% paraformaldehyde in PBS at 4°C for up to 1 week. Fixed blastocysts were washed three times for 5 min each in PBS containing 0.1% BSA and transferred to PBS containing 0.5% Triton X-100 (Sigma-Aldrich, Slovakia) for 1 h. After permeabilization, the blastocysts were washed twice for 15 min each in PBS containing BSA and were subsequently incubated in the TUNEL assay reagents (*In Situ* Cell Death Detection Kit; Roche, Penzberg, Germany) for 1 h at 37°C in the dark. Non-specific immunoreactions were blocked using 10% normal goat serum (Santa Cruz Biotechnology, Santa Cruz, CA, USA) for 2 h at room temperature. After blocking, the mouse blastocysts were incubated with primary antibody (rabbit anti-mouse CDX2 polyclonal antibody, 1:100 dilution; Cell Signaling Technology, Danvers, MA, USA) diluted in blocking solution at 4°C overnight. The next day, the blastocysts were washed twice in the blocking solution and incubated with Texas-Red-X goat anti-rabbit IgG secondary antibody (1:200 dilution; Jackson ImmunoResearch Laboratories, West Grove, PA, USA) for 1 h. Finally, the blastocysts were counterstained with Hoechst 33342 (10 µL/mL in PBS; Sigma-Aldrich) for 5 min at room temperature, mounted on glass slides using Vectashield (Vector Laboratories, Burlingame, CA, USA) and observed using a fluorescence microscope at magnification ×400 (BX51; Olympus, Tokyo, Japan).

According to their nuclear morphology (M) and TUNEL assay positivity/negativity (T±), dead cells were classified as follows: (1) typical apoptotic cells: MaT+, with fragmented (occasionally pycnotic) nuclear morphology and TUNEL-positive nucleoplasm; MaT−, with fragmented nuclear morphology and TUNEL-negative nucleoplasm; or MnT+, with normal nuclear morphology and TUNEL-positive nucleoplasm. (2) Other dead cells: MkT−/+, with abnormal (karyolysis-like) nuclear morphology and occasional TUNEL-positive labeling. In each blastocyst, the percentage of dead cells (calculated as the number of dead cells relative to the total number of blastomeres in blastocyst or in particular cell line) and the frequency of particular types of dead cells (calculated as the number of apoptotic or other dead cells relative to the total number of blastomeres in blastocyst) were evaluated.

### Real-time RT-PCR Analysis of the Selected Transcripts in *In Vivo* Developed Blastocysts

Total RNA was extracted from batches of 50 to 100 mouse blastocysts using TRIzol Reagent (Invitrogen Life technologies, Karlsruhe, Germany) according to the manufacturer’s instructions. Contaminating DNA in the RNA preparations was digested with amplification-grade DNase I (Invitrogen Life Technologies). For the quantitative analysis, 0.2 pg of luciferase (Luc) mRNA (Promega, Madison, WI, USA) per blastocyst was added to the TRIzol lysis reagent prior to the RNA extraction to correct differences in RNA recovery and loading of RT-PCRs. Six separate batches of blastocysts were used for the RNA isolation and cDNA synthesis in each experimental group.

The RNA was reverse transcribed using Superscript™ III RNase H− Reverse Transcriptase (Invitrogen Life Technologies) and a mixture of anchored oligo-dT primers and random hexamers (Thermo Fisher Scientific—ABgene, Waltham, MA, USA). To detect the presence of genomic DNA contamination in the RNA preparations, reverse transcriptase negative controls (no reverse transcriptase in the reaction) were performed in parallel, using a part of each RNA sample. The cDNA preparations were subsequently cleaned via ethanol precipitation, and the cDNA pellets were diluted in an appropriate amount of 10 mM Tris (pH 8.3) so that 1 µL of the cDNA corresponded to 2.5 blastocyst (and 0.5 pg Luc mRNA) equivalents.

PCR amplifications were performed in an Mx 3000P real-time PCR system (Stratagene, La Jolla, CA, USA). The reactions were performed in 25-µL reaction volumes containing 1 µL of cDNA, 1× SYBR Green/ROX PCR mix (Qiagen, Valencia, CA, USA), and 0.4 µM of specific primers (Table 3). An initial denaturation step at 95°C for 10 min was followed by 40 to 50 cycles at 95°C for 20 s, annealing at the primer-specific temperature for 30 s and elongation at 72°C for 20 s (Table 3). For luciferase amplification, we used primers designed in a previous study (24). An initial step at 95°C for 10 min was followed by 40 cycles at 95°C for 20 s, 65°C for 30 s, and 72°C for 20 s.

**Table 3:** Commercial primer sets used for real-time PCR analysis.

Gene	GenBank no.	Primers	Amplicon size (bp)	Ta	No. c.
*BAX*	NM_007527.3	PPM02917E	105	65	40
*BCL2L2*	NM_007537.1	PPM02919A	92	65	40
*PLIN2*	NM_007408.3	PPM05362F	121	68	40
*SLC27A4 (FATP4)*	NM_011989.4	PPM03287A	123	68	40
*LEPR*	NM_010704.2	PPM05512A	83	68	40
*SLC2A4 (GLUT4)*	NM_009204.2	PPM04166F	89	65	45
*LEP*	NM_008493.3	PPM03504B	63	60	50

*Primers (catalog numbers of RT2 qPCR Primer Assays, Qiagen), size of amplicons (in base pairs, bp), annealing temperature (Ta, °C), and number of amplification cycles (no. c.) used in PCR are shown*.

Fluorescence was measured after the elongation step. Amplification specificity was assessed with a melting curve analysis. The expression of the target genes was normalized to the external control: luciferase mRNA in each sample [i.e., the mRNA relative quantity of a target gene was divided by the Luc mRNA relative quantity (25)]. The fluorescence data obtained from the amplifications were transformed to values of relative mRNA quantity using two methods of analysis: the relative standard curve method (using serial dilutions of mouse genomic DNA or luciferase cDNA) and the LinRegPCR-Ct method [fitting log-transformed fluorescence data from the exponential PCR phase with the line (24)]. Both methods showed similar results, indicating their reliability. The quantification results obtained using the relative standard curve method are presented.

### Statistical Analysis

Statistical analysis was performed using GraphPad Prism Software 5.01 (GraphPad Software, Inc., La Jolla, CA, USA). The results are expressed as the mean values ± SEM.

Differences between data showing normal Gaussian distribution were assessed using standard parametric tests, i.e., Student’s *t*-test or ANOVA, followed by Tukey’s *post hoc* test, depending on the number of evaluated groups. This analysis involved the following parameters: body weights of female mice, plasma concentrations of leptin, the mean number of blastomeres per blastocyst, and the expression profiles of particular genes in blastocysts.

The differences between data that did not pass normality tests were assessed using the Mann–Whitney test or the Kruskal–Wallis test, followed by Dunn’s *post hoc* test, depending on the number of evaluated groups. This analysis involved the following parameters: amount of body fat in mice females, the number of isolated embryos per dam, the capacity of embryos to reach a particular embryonic stage *in vitro* considering drop-to-drop variability, the ICM/TE ratio in blastocysts, cell death incidence per blastocyst and per particular cell lineage (ICM, TE), and the incidence of particular morphological features in dead cells.

To assess differences between score-type data, standard chi-square tests with one (the proportion of fertilized embryo donors, the proportion of isolated two-cell embryos, the distribution of dead cells between ICM and TE lines) or three degrees of freedom (overall developmental capacities of embryos *in vivo* and *in vitro*) were used.

Differences of *P* < 0.05 were considered statistically significant.

## Results

### Development of Two-Cell Embryos from Control and Obese Dams *In Vitro*

During the reproductive study, more than 50% of females of both condition types underwent spontaneous ovulation and were naturally fertilized (Tables 1 and 2). As shown in Table 1, there was no difference in the average number of embryos isolated from control and obese females on day 2 of pregnancy (*P* > 0.05). In both groups, approximately 90% of isolates represented embryos at the two-cell stage, while the remaining isolates were non-cleaving or degenerated oocytes.

**Figure 2:**
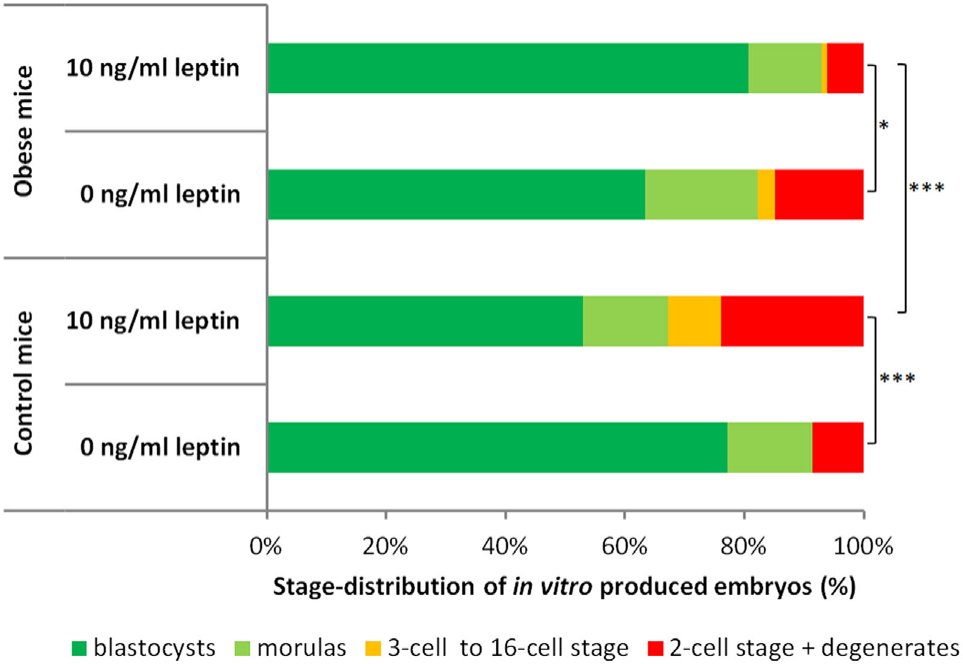
Developmental capacities of two-cell embryos isolated from control and obese mice and cultured for 72 h *in vitro*. The graph shows the proportion (%) of arrested embryos (two-cell stage and degenerates) and embryos that reached higher developmental stages (3- to 16-cell stage, morulas and blastocysts). Embryos from control mice were cultured in the leptin-free media (*n* = 105) or media supplemented with leptin at 10 ng/mL (*n* = 134). Embryos from obese mice were cultured in the leptin-free media (*n* = 101) or media supplemented with leptin at 10 ng/mL (*n* = 98). Asterisks indicate statistical differences: chi-square test with three degrees of freedom, *P < 0.05; ***P < 0.001.

As shown in Figure 2, the two-cell embryos isolated from obese mice females and cultured for 72 h in leptin-free media showed overall developmental capacities similar to their counterparts isolated from control mice (chi-square test, *P* = 0.07).

The presence of leptin at 10 ng/mL had a significant effect on embryonic development and differentially influenced embryos isolated from control and obese females (Figure 2). Leptin negatively affected the overall development of embryos isolated from control females (*P* < 0.001): leptin significantly increased the proportion of embryos arrested at the two-cell stage or that had degenerated (*P* < 0.05) and decreased the proportion of embryos reaching the blastocyst stage (*P* < 0.05). In contrast, leptin showed a beneficial effect on the development of embryos isolated from obese females (*P* < 0.05): leptin significantly decreased the proportion of embryos arrested at the two-cell stage or that had degenerated (*P* < 0.05).

As shown in Table 4, there was no significant difference in the majority of qualitative parameters evaluated in blastocysts obtained *in vitro* from two-cell embryos isolated from either obese females or controls and cultured in leptin-free media, i.e., in the mean number of cells per blastocyst, cell differentiation into ICM and trophectoderm, and the average percentage of dead cells per blastocyst (*P* > 0.05 for all cases). The only differences were observed in the distribution of dead cells between ICM and TE lines (*P* < 0.05) and the average percentage of dead cells in the trophectoderm (*P* < 0.05); the TE cell line of blastocysts derived from obese females showed significantly lower occurrence and incidence of dead cells.

**Table 4:** Qualitative parameters of *in vitro*-obtained blastocysts originating from control and obese mice.

	Control mice		Obese mice	
	0 ng/mL leptin	10 ng/mL leptin	0 ng/mL leptin	10 ng/mL leptin
Number of evaluated blastocysts (*n*)	72	63	60	69
Mean number of cells per blastocyst	67.78 ± 2.06	71.13 ± 2.33	63.65 ± 2.06	67.30 ± 2.28
Cell differentiation (% ICM:TE cells)	34.37:65.63	29.27:70.73	34.63:65.36	34.60:65.40
ICM/TE ratio	0.56 ± 0.03	0.44 ± 0.04	0.58 ± 0.06	0.59 ± 0.05
Average % of dead cells in blastocysts	6.23 ± 0.54^a^	5.89 ± 0.59^a,b^	6.14 ± 0.54^a^	4.15 ± 0.35^b^
Distribution of dead cells (% in ICM:TE)	88.46:11.54	84.91:15.09	99.11:0.89^a^	93.92:6.08^a^
Average % of dead cells in ICM line	18.43 ± 1.90^a^	18.22 ± 1.86^a^	21.71 ± 3.62^a^	11.74 ± 1.15^b^
Average % of dead cells in TE line	1.51 ± 0.43^a^	1.37 ± 0.29^a^	0.14 ± 0.09^b^	0.50 ± 0.15^b^

*The results are expressed as the mean values ± SEM. Different letters in superscript indicate statistical differences; mean number of cells: ANOVA followed by Tukey’s test; ICM/TE ratio, average % of dead cells in blastocysts, in ICM line, and in TE line: Kruskal–Wallis followed by Dunn’s test; the distribution of dead cells between ICM and TE lines: chi-square test*.

Embryos isolated from control and obese dams and cultured in medium supplemented with leptin produced blastocysts of different qualities (Table 4). In the case of control females, the qualitative parameters in the obtained blastocysts were comparable to those evaluated in blastocysts cultured in leptin-free media. In the case of obese females, blastocysts obtained from embryos treated with leptin contained a significantly lower percentage of dead cells than blastocysts obtained in leptin-free media (*P* < 0.05). This decrease was predominantly evident in the ICM cell line (*P* < 0.05).

**Figure 3:**
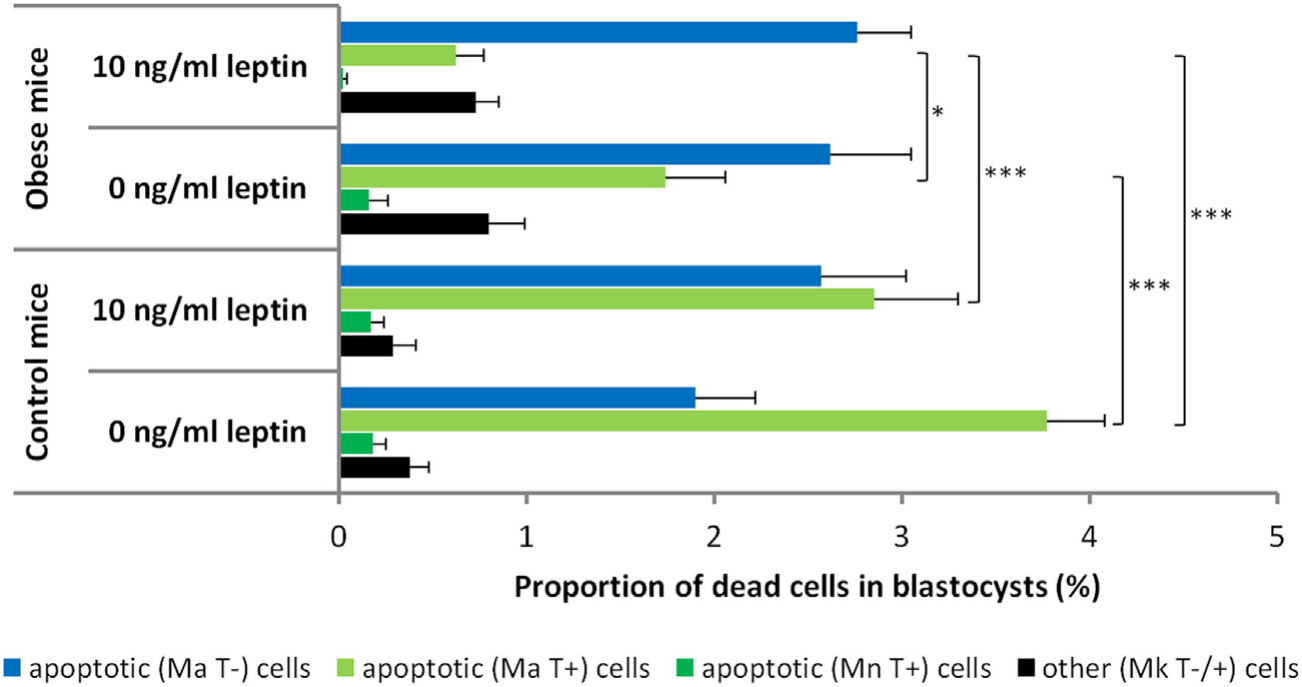
Proportion of dead cells in blastocysts obtained from the *in vitro* culture of the two-cell embryos isolated from control and obese mice. According to the presence of particular morphological features [normal nuclear morphology (Mn), nuclear fragmentation or condensation (Ma), nuclear karyolysis (Mk), and positive/negative terminal deoxynucleotidyl transferase dUTP nick end labeling (TUNEL) labeling (T±)], the dead cells were classified as follows: (1) apoptotic, showing fragmented nuclear morphology or TUNEL-positive nucleoplasm or both or (2) other dead cells, showing karyolysis-like nuclear morphology and occasional TUNEL labeling. Embryos from control and obese mice were cultured in leptin-free media or media supplemented with leptin at 10 ng/mL. Numbers of evaluated blastocysts are shown in Table 4. The results are expressed as the mean values + SEM. Asterisks indicate statistical differences between control and obese groups: Kruskal–Wallis test, followed by Dunn’s test, *P < 0.05; ***P < 0.001.

Morphological analysis revealed the predominantly apoptotic origin of dead cells in blastocysts in all experimental groups. These cells showed the presence of nuclear fragmentation (occasionally pycnotic condensation) or specific DNA degradation in the nucleoplasm visualized *via* TUNEL labeling or both. Interestingly, when the occurrence of particular apoptotic features was analyzed, blastocysts originating from control mice predominantly contained dead cells displaying nuclear fragmentation accompanied with TUNEL-positive labeling, and blastocysts originating from obese mice predominantly contained dead cells displaying nuclear fragmentation without TUNEL labeling (Figure 3).

The results of parallel culture experiments showed that the presence of leptin solvent did not affect the developmental capacities of the two-cell embryos or the quality of *in vitro*-obtained blastocysts (Datasheet S1 in Supplementary Material).

### Gene Expression in *In Vivo*-Developed Blastocysts Isolated from Control and Obese Dams

Analysis of the blood samples randomly collected from 18 fertilized mice on day 4 of pregnancy confirmed previous results showing higher plasma concentrations of leptin in obese females delivered from dams fed a high-energy diet than in controls delivered from dams fed a standard diet (2.09 ± 0.19 vs. 1.25 ± 0.10 ng/mL, *P* < 0.01).

As shown in Table 2, there was no difference in the average number of embryos isolated from control and obese females on day 4 of pregnancy (*P* > 0.05). On the other hand, embryos isolated from obese females showed significantly retarded development represented by decreased numbers of isolated blastocysts and morulas (87.58 vs. 91.49%) and increased numbers of slowly developing embryos and degenerates (12.42 vs. 8.50%; *P* < 0.001).

**Figure 4:**
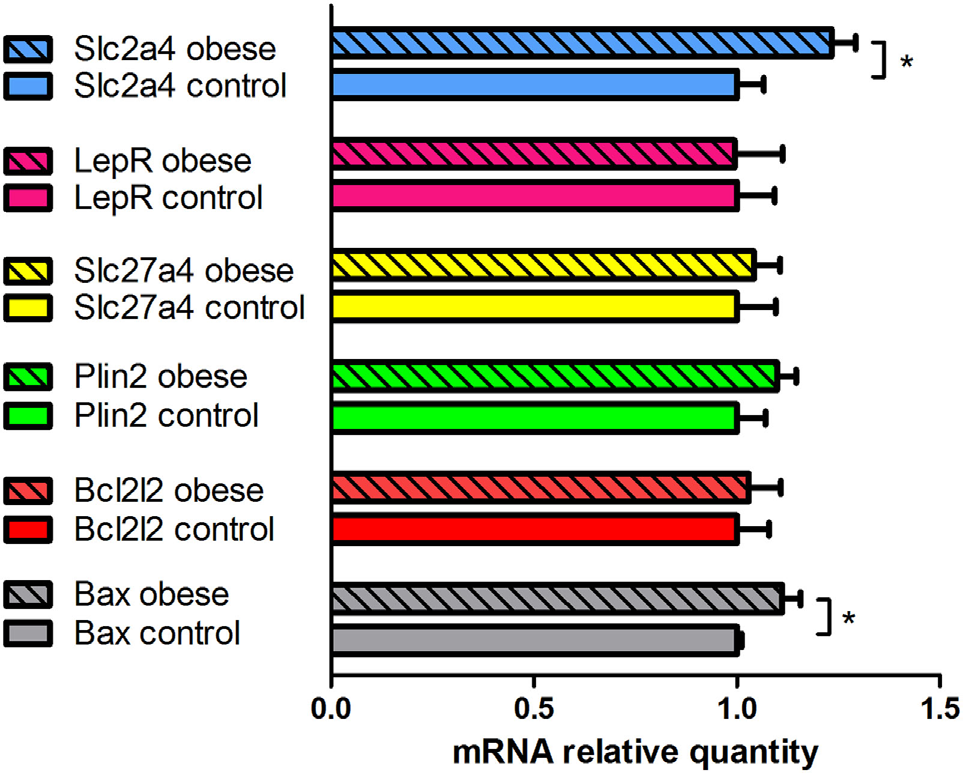
Quantitative analysis of mRNA expression of selected genes in *in vivo-*produced blastocysts obtained from control and obese female mice. The expression of the target genes was normalized to the external control (luciferase mRNA) in each sample: the mRNA relative quantity of a target gene was divided by the Luc mRNA relative quantity. The results are expressed as the mean values + SEM (*n* = 6). The mean values obtained for the control group (open columns) were set to 1.0, and the mean values obtained for the obese group (full columns) are expressed relative to the control group. An asterisk indicates a significant difference: Student’s *t*-test, *P < 0.05.

RT-PCR analysis showed that mouse blastocysts isolated from both groups of donors expressed quantifiable amounts of mRNA of the following genes: *BAX*, encoding a proapoptotic protein of the Bcl-2 family; *BCL2L2*, encoding an anti-apoptotic protein of the Bcl-2 family; *LEPR*, encoding the leptin receptor; *PLIN2* (*ADFP*), encoding perilipin 2 (adipophilin); *SLC27A4* (*FATP4*), encoding fatty acid transporter protein 4; and *SLC2A4* (*GLUT4*) encoding facilitated glucose transporter protein 4 (Figure 4). Leptin expression was markedly low, and no PCR product was detected in some cDNA samples [a weak leptin PCR product was obtained in 58.33% (14/24) of the cDNA samples analyzed; the leptin expression was distributed randomly between experimental groups].

Statistical analysis showed that blastocysts isolated from obese female mice displayed significantly higher expression of *BAX* and *SLC2A4* (*GLUT4*) than blastocysts isolated from control mice (*P* < 0.05). These cells also displayed a slightly higher *BAX*/*BCL2L2* ratio (*P* = 0.05). A similar tendency was observed for the *PLIN2* gene, although without statistical significance (*P* > 0.05). *LEPR* and *SLC27A4* (*FATP4*) expression levels were similar in both groups of blastocysts.

## Discussion

### Role of Leptin in Preimplantation Development

In the present study, we observed leptin mRNA expression in mouse blastocysts, in contrast with the results of Herrid et al. (12), which claimed the absence of leptin expression. Kawamura et al. (26) did not detect leptin mRNA in mouse blastocysts using standard RT-PCR, and reamplification (nested PCR) was required to obtain a consistent signal. Taken together, the results indicate that leptin expression in mouse blastocysts is apparently low; inconsistencies in the detection of this low-copy mRNA could reflect stochastic gene expression or transcriptional “leakage” (27, 28). However, appreciable leptin secretion has been reported in equine (29), swine (30, 31), and human (32) blastocysts.

Previously, expression of three isoforms of the leptin receptor, including the most important isoform (LeptRb) responsible for signal transduction, has been documented in mouse preimplantation embryos at all stages of development (from the two-cell to the blastocyst) at both mRNA and protein levels (5, 12, 16, 26). Similar observations have been reported in ovine (12), bovine (33), swine (30), and human embryos (32). Herein, we also confirmed leptin receptor expression in mouse blastocysts.

The ability of leptin to influence the development of preimplantation embryos through its receptor has been evaluated in several *in vitro* studies. When mouse or swine preimplantation embryos were cultured with leptin at 1 (or 1.6) ng/mL, no effect was observed on their developmental potential or cell proliferation (9, 12, 16, 26, 34). Thus, we concluded that embryonic cells do not respond to the presence of physiological levels of leptin.

When culture media were supplemented with leptin at 10 (or 16) ng/mL, some authors reported no effect [mouse (16, 35); pig (36, 37)], while others have reported positive effects [mouse (9, 12, 26); pig (34)] on preimplantation embryo development. In two cases, an increase in the total cell number in blastocysts was documented [mouse (12); pig (34)]. Except for a slight increase in apoptotic index observed after the addition of leptin at 16 ng/mL in one mouse experiment (16), no studies have reported a negative effect of 10 ng/mL leptin on cultured embryos. Moreover, a reduced incidence of TUNEL-positive blastomeres was documented in bovine blastocysts derived from oocytes matured in the presence of leptin at 10 ng/mL (17).

Even more conflicting results were obtained after the use of higher, physiologically unreachable concentrations of this hormone (100 to 1,000 ng/mL): some studies reported no effect [mouse 100 ng/mL (35); mouse 160 ng/mL (16)], while other studies reported a negative effect [mouse 100 ng/mL (12); pig 500 ng/mL (36, 37)], and yet other studies reported a positive effect [mouse 100 and 1,000 ng/mL (9, 26); pig 100 ng/mL (37)] of leptin on the development and proliferative activity of preimplantation embryos.

The differences between these results (e.g., positive vs. no effect at a concentration of 10 ng/mL) might reflect the use of a different type of recombinant leptin (human vs. mouse), a different origin of embryos (naturally fertilized vs. IVF or parthenogenetically activated oocytes), a different culture time with leptin (starting from the two-cell stage vs. the zygote stage) or the specificity of the species/strain. However, these variations cannot clarify the highly contradictory results, such as positive vs. negative effects at high (>100 ng/mL) concentrations. Thus, other factors typically not evaluated in these experiments, such as the potential toxicity of the leptin solvent used, the composition of the media used (known/unknown, with/without amino acids), or, as shown in the present study, the condition of the embryo donors, should be considered.

In the present study, two-cell embryos isolated from control and obese female mice were cultured in synthetic oviductal medium supplemented with leptin at a concentration of 10 ng/mL. Embryos isolated from control female mice showed retarded development after leptin treatment. No impact on apoptosis incidence was observed in these embryos. In the case of embryos isolated from obese females, a positive influence of elevated leptin concentration was observed: the two-cell embryos of such origin showed a better developmental capacity *in vitro*, and the obtained blastocysts contained significantly lower numbers of apoptotic cells on average.

Since the results obtained in embryos isolated from obese mice are consistent with the outcomes of a majority of studies assessing the effect of leptin (10 ng/mL) on preimplantation embryos *in vitro* (9, 12, 17, 26), we hypothesized that the embryo donors in these studies either consumed diets with higher caloric value or displayed elevated amounts of body fat. There is a long-term trend followed by the commercial sphere to design and produce diets “better supporting growth and reproduction of rodents,” which typically contain higher percentages of crude fat (4.5 to 9%) than traditional diets formulated decades ago (such as the M1 diet used in the present study containing only 2.7% crude fat). The consumption of such diets does not necessarily lead to overweight mice; however, a high-fat diet is typically accompanied by higher accumulation of abdominal fat (an observation made in our animal breeding house, not published data) and the potential elevation of plasma leptin.

The question remains: why do *in vitro*-cultured embryos from control and obese females respond differently to leptin? The answer might be that the germ (or early embryonic) cells of obese females adapt to elevated levels of leptin during maturation (similar to somatic cells developing resistance to leptin or insulin), and this adaptation positively influences the response to subsequent hormone exposure. However, non-adapted oocytes (developed in control mice) responded negatively to the sudden elevation of leptin, i.e., a higher proportion of these embryos arrested at the two-cell (or following) stage.

The mechanism of this hypothesized adaptation is unknown. Since studies have previously shown that bovine day 7 blastocysts derived from oocytes or embryos cultured *in vitro* in the presence of 10 ng/mL of leptin display significant changes in *LEPR* expression [decreased or increased, depending on the period of exposure to leptin (33)], we proposed that this mechanism is based on the regulation of leptin receptor expression. However, we did not observe any differences between *LEPR* expression in blastocysts isolated from control and obese mice that were exposed to elevated levels of leptin during both oocyte maturation and embryo development *in vivo*, consistent with previous *in vitro* results showing unaffected *LEPR* expression in bovine blastocysts exposed to leptin during both oocyte and embryo development (33). Thus, this regulation might also occur at the cytoplasmic or nuclear level (11).

Epigenetic modifications likely represent the most important item of a hypothesized adaptation mechanism. The results of recent experimental studies have shown that numerous environmental factors, including maternal obesity, might significantly change the DNA methylation of various genes in developing oocytes and embryos (38, 39). In our transgenerational obesity model, the presence of overnutrition during intrauterine and early postnatal development would induce epigenetic modifications even in the primordial germ cells of experimental animals. As hypothesized previously, the genome-wide reprograming of the primordial germ cells which starts at about mid-way through gestation might represent an important window of environmental sensitivity (40). According to the “hypothesis of developmental origins of adult diseases” (41), such genomic changes might have significant impacts on the further development of the conceptus or the health of the offspring. A decrease in body weight and fat in mouse offspring delivered from obese dams, shown in our previous study (14), might also be correlated with such changes.

### Apoptosis and Gene Expression in Blastocysts Recovered from Control and Obese Dams

In our previous studies, *in vivo* developed blastocysts isolated from obese mice females (delivered by overfed dams in the same outbred model) displayed increased apoptosis (13). These results were confirmed in the present study: we documented significantly elevated expression of the proapoptotic regulatory gene *BAX* in blastocysts isolated from obese female mice. As previously hypothesized, the increased incidence of apoptosis likely reflects higher concentrations of apoptotic inductors in the embryonic microenvironment or the elimination of a higher number of embryonic cells with undefined subcellular damage. This hypothesis is consistent with the fact that the elevated accumulation of body fat in mothers is accompanied by metabolic and humoral changes, leading to the production of numerous biological agents that directly (cytokines) or indirectly (hyperglycemia) trigger apoptosis in various tissues, including the reproductive tract [reviewed in Ref. (42, 43)].

Still, it is not known if the increased incidence of cell death in blastocysts results from changes in the embryo environment during the preimplantation period or if increased cell death is the consequence of lower quality oocytes produced by obese females, as documented in other studies (14, 44–46). Since the embryos isolated from control and obese female mice cultured under identical conditions (leptin-free groups in the present study) showed no significant differences in developmental capacities or quality (including frequency of apoptosis), we proposed that maternal overweight should be considered a risk factor, even during the embryo preimplantation period.

Nevertheless, although the frequencies of apoptotic processes were similar, some differences between *in vitro*-cultured blastocysts from control and obese dams were observed in terms of the localization and morphology of the dead cells: in blastocysts produced from the two-cell embryos isolated from obese female mice, significantly lower occurrence and incidence of dead cells in the trophectoderm cell line and lower frequency of specific DNA degradation (TUNEL labeling) in the nuclei of cells with typical features of apoptosis were recorded. This finding might suggest some differences in the mechanisms through which blastomeres with detrimental potential are eliminated in embryos originating from obese dams.

The results of previous studies evaluating the relationship between obesity and reproduction have suggested that maternal overweight status might affect the metabolic activity of preimplantation embryos (42, 43, 47). To support this hypothesis, we compared the expression profiles of two genes associated with fat metabolism: perilipin 2 and fatty acid transporter protein 4. The elevated expression levels of these genes were previously documented in blastocysts isolated from obese female rabbits fed a hyperlipidic hypercholesterolemic diet (48) and inbred New Zealand Obese mice (49). Although perilipin 2 expression was higher in blastocysts isolated from obese mice than in those isolated from control mice, statistical analysis did not confirm a significant difference. Apparently, broader gene expression analyses are required in the future, that consider potential species-specific differences and the model of production of obese females.

However, when we examined the expression of a member of the facilitative glucose transporter family, *SLC2A4* [*GLUT4*, an insulin-responsive glucose transporter (50)], we observed the upregulation of the transporter in blastocysts isolated from obese females. Previously, in blastocysts recovered from diet-induced obese females, *SLC2A1* (*GLUT1*, a glucose transporter responsible for basal glucose uptake) down-regulation was demonstrated (51), whereas in mouse blastocysts conceived by obese females and obese males (“combined obese parented embryos”), *SLC2A1* upregulation was observed (52). These results indicate that both maternal and paternal obesity can induce different changes in the expression profiles of particular members of the facilitative glucose transporter family in blastocysts. The elevated expression of *SLC2A4* (*GLUT4)* detected in the present study might correlate with elevated levels of insulin and glucose in the plasma of obese female mice developed in this two-generation model and documented in previous studies (13, 19).

## Conclusion

We conclude that elevated levels of leptin have both positive and negative effects on preimplantation embryo development *in vitro*, and this response likely depends on the previous exposure of germ cells to the presence of the hormone in the environment. Concerning the potential role of leptin in embryonic cell death regulation, at 10 ng/mL, leptin acts as a survival factor rather than an apoptotic inductor. Since no elevations in the expression levels of the leptin receptor or fat metabolism-associated genes were recorded in blastocysts recovered from obese female mice, the role of leptin in mediating the effects of obesity on embryos at the peripheral level is likely lower than expected. The observed upregulation of the insulin-responsive glucose transporter indicates that insulin might be a more important mediator.

## Ethics Statement

All animal experiments were performed in accordance with the ethical principles of the Ethical Committee for Animal Experimentation at the Institute of Animal Physiology, approved through the State Veterinary and Food Administration of the Slovak Republic (Ro 2296/13-221c) in strict accordance with Slovak legislation based on EU Directive 2010/63/EU on the protection of animals used for experimental and other scientific purposes. All efforts were made to reduce animal suffering and distress and to minimize the number of animals necessary to produce reliable results.

## Author Contributions

All authors contributed to the acquisition, analysis and interpretation of data, manuscript drafting, and critical discussions on its content. DF was responsible for work design and manuscript writing. All authors revised and accepted the final version of manuscript.

## Conflict of Interest Statement

The authors declare that the research was conducted in the absence of any commercial or financial relationships that could be construed as a potential conflict of interest.
