# Supplementary material for: The Responses of Mouse Preimplantation Embryos to Leptin In Vitro in a Transgenerational Model for Obesity
Source: Front Endocrinol (Lausanne). 2017 Sep 13;8:233. doi: 10.3389/fendo.2017.00233 (PMC5604062; doi:10.3389/fendo.2017.00233)

Supplementary Material

The Responses of Mouse Preimplantation Embryos to Leptin In Vitro in a Transgenerational Model for Obesity

Martina Kšiňanová, Štefan Čikoš, Janka Babeľová, Zuzana Šefčíková, Alexandra Špirková, Juraj Koppel and Dušan Fabian*

*** Correspondence:** Dušan Fabian: fabian@saske.sk

# Supplementary Data

To test potential toxicity of leptin’s solvent, parallel culture experiments with the 20 mM TRIS/HCl (0.001% v/v) were provided.

Embryos at the 2-cell stage isolated from dams of each condition type (control and obese) were cultured *in vitro* under standard conditions (a humidified atmosphere with 5% CO2 and 37°C) for 96 h with / without the presence 20 mM TRIS/HCl (Sigma-Aldrich, Bratislava, Slovakia). To provide an appropriate developmental environment (i.e., one embryo per at least 1 µl of medium), 20 to 25 embryos were placed into 30 µl of synthetic oviductal medium KSOMaa Evolve containing an elevated potassium concentration, half-strength Eagle non-essential amino acid mixture (Zenith Biotech, Canada) and 0.1% (w/v) bovine serum albumin (BSA, Sigma-Aldrich). Drops of medium were placed in plastic cell culture dishes and covered with mineral oil (Zenith Biotech). The final concentration of 20 mM TRIS/HCl in KSOMaa was 0.01 μl/ml.

The results of parallel culture experiments showed that the presence of leptin solvent did not affect the developmental capacities of the 2-cell embryos (**Figure 5**) or the quality of *in vitro*-obtained blastocysts (**Table 5**, **Figure 6**). The embryos were almost identical to those observed in control (leptin-free and TRIS/HCl-free) groups.

# Supplementary Figures and Tables

## Supplementary Tables

**Table 5. Qualitative parameters of *in vitro*-obtained blastocysts originating from control and obese mice.**

|  | **Control mice** | | **Obese mice** | |
| --- | --- | --- | --- | --- |
|  | **- TRIS/HCl** | **+ TRIS/HCl** | **- TRIS/HCl** | **+ TRIS/HCl** |
| **Number of evaluated blastocysts (n)** | 72 | 46 | 60 | 45 |
| **Mean number of cells per blastocyst** | 67.78±2.06 | 67.35±2.85 | 63.65±2.06 | 68.78±2.57 |
| **Cell differentiation**  **(% ICM:TE cells)** | 34.37 : 65.63 | 37.25 : 62.75 | 34.63 : 65.36 | 32.31 : 67.69 |
| **ICM/TE ratio** | 0.56±0.03 | 0.63±0.04 | 0.58±0.06 | 0.51±0.04 |
| **Dead cells in blastocysts** | 6.23±0.54% | 6.30±0.67% | 6.14±0.54% | 6.67±0.57% |
| **Distribution of dead cells (% in ICM:TE)** | 90.88 : 9.12 | 87.61 : 12.39 | 98.64 : 1.36 | 97.10 : 2.90 |
| **Dead cells in ICM line** | 18.43±1.90% | 18.73±3.53% | 21.71±3.62% | 22.36±2.36% |
| **Dead cells in TE line** | 1.51±0.43%^a^ | 0.67±0.17%^a,b^ | 0.14±0.09%^b^ | 0.24±0.11%^a,b^ |

The results are expressed as the mean values ±SEM. Different letters in superscript indicate statistical differences; mean number of cells: ANOVA followed by Tukey’s test; ICM/TE ratio, proportion of dead cells in blastocysts, dead cells in ICM, dead cells in TE: Kruskal-Wallis followed by Dunn’s test.

## Supplementary Figures

**Figure 5. Developmental capacities of 2-cell embryos isolated from control and obese mice and cultured for 96 h *in vitro*.** The graph shows the proportion (%) of arrested embryos (2-cell stage and degenerates) and embryos that reached higher developmental stages (3 to 16-cell stage, morulas and blastocysts). Embryos from control mice were cultured in the culture media with (n=99) / without (n=105) the presence of TRIS/HCl. Embryos from obese mice were cultured in the culture media with (n=163) / without (n=101) the presence of TRIS/HCl. Differences were assessed using chi-square test with three degrees of freedom (P>0.05 for all cases).


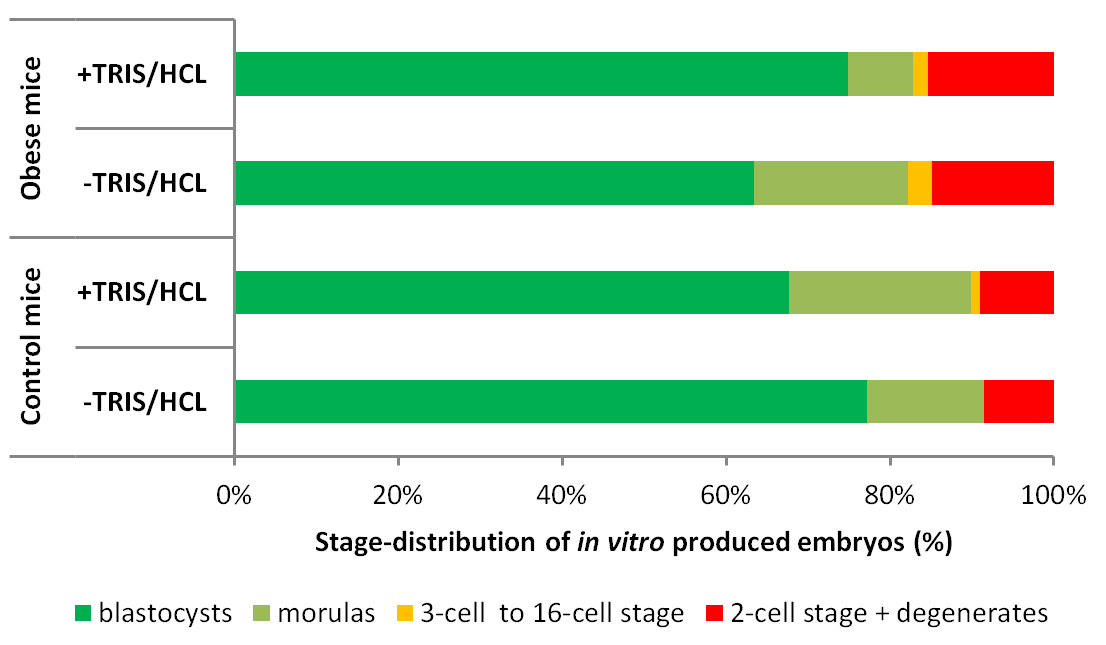


**Figure 6. Proportion of dead cells in blastocysts obtained from the *in vitro* culture of the 2-cell embryos isolated from control and obese mice.** According to the presence of particular morphological features [normal nuclear morphology (Mn), nuclear fragmentation or condensation (Ma), nuclear karyolysis (Mk), and positive/negative TUNEL labeling (T+/-)], the dead cells were classified as follows: 1. Apoptotic, showing fragmented nuclear morphology or TUNEL-positive nucleoplasm or both; or 2. Other dead cells, showing karyolysis-like nuclear morphology and occasional TUNEL labeling. Embryos from control and obese mice were cultured in the culture media with / without the presence of TRIS/HCl. Differences were assessed using the Kruskal-Wallis test, followed by Dunn’s test. Asterisks indicate statistical differences between control and obese groups: * P<0.05; ***P<0.001.


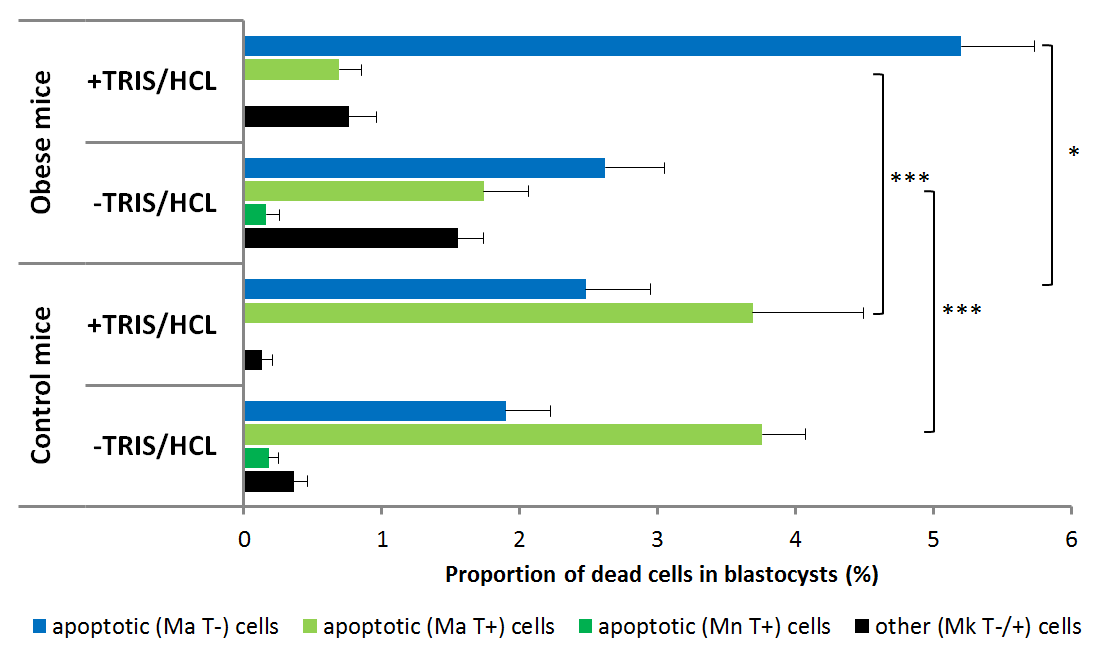

Supplement: Supplementary file 1 [file Data_Sheet_1.DOCX]
